# Estimating Maximal Oxygen Uptake From Daily Activity Data Measured by a Watch-Type Fitness Tracker: Cross-Sectional Study

**DOI:** 10.2196/13327

**Published:** 2019-06-13

**Authors:** Soon Bin Kwon, Joong Woo Ahn, Seung Min Lee, Joonnyong Lee, Dongheon Lee, Jeeyoung Hong, Hee Chan Kim, Hyung-Jin Yoon

**Affiliations:** 1 Interdisciplinary Program in Bioengineering Seoul National University Seoul Republic of Korea; 2 Institute of Medical & Biological Engineering Medical Research Center Seoul National University Seoul Republic of Korea; 3 Department of Biomedical Engineering Seoul National University College of Medicine Seoul Republic of Korea; 4 Biomedical Research Institute Seoul National University Hospital Seoul Republic of Korea

**Keywords:** cardiorespiratory fitness, oxygen consumption, fitness tracker

## Abstract

**Background:**

Cardiorespiratory fitness (CRF), an important index of physical fitness, is the ability to inhale and provide oxygen to the exercising muscle. However, despite its importance, the current gold standard for measuring CRF is impractical, requiring maximal exercise from the participants.

**Objective:**

This study aimed to develop a convenient and practical estimation model for CRF using data collected from daily life with a wristwatch-type device.

**Methods:**

A total of 191 subjects, aged 20 to 65 years, participated in this study. Maximal oxygen uptake (VO_2_ max), a standard measure of CRF, was measured with a maximal exercise test. Heart rate (HR) and physical activity data were collected using a commercial wristwatch-type fitness tracker (Fitbit; Fitbit Charge; Fitbit) for 3 consecutive days. Maximal activity energy expenditure (aEEmax) and slope between HR and physical activity were calculated using a linear regression. A VO_2_ max estimation model was built using multiple linear regression with data on age, sex, height, percent body fat, aEEmax, and the slope. The result was validated with 2 different cross-validation methods.

**Results:**

aEEmax showed a moderate correlation with VO_2_ max (*r*=0.50). The correlation coefficient for the multiple linear regression model was 0.81, and the SE of estimate (SEE) was 3.518 mL/kg/min. The regression model was cross-validated through the predicted residual error sum of square (PRESS). The PRESS correlation coefficient was 0.79, and the PRESS SEE was 3.667 mL/kg/min. The model was further validated by dividing it into different subgroups and calculating the constant error (CE) where a low CE showed that the model does not significantly overestimate or underestimate VO_2_ max.

**Conclusions:**

This study proposes a CRF estimation method using data collected by a wristwatch-type fitness tracker without any specific protocol for a wide range of the population.

## Introduction

Cardiorespiratory fitness (CRF) is an important component of physical fitness, representing the body’s ability to take oxygen in and deliver this oxygen to muscle cells throughout the body during physical activity. Previous studies have emphasized the importance of CRF, providing convincing evidence that CRF is closely related to all-cause mortality [1,2]. In addition, CRF is known to be correlated with various physiological factors, such as body composition and blood pressure, and psychological factors, such as depression [1-4]. Erikssen et al [5] have reported that a change in physical fitness is a strong predictor of mortality. They found that a small improvement in physical fitness can significantly lower the risk of death.

Maximal oxygen uptake (VO_2_ max) is regarded as a representative feature of CRF. The current gold standard for measuring VO_2_ max is a metabolic gas analysis during a maximal graded exercise test (GXT) on a treadmill or other equipment, such as cycle ergometer. Even though the maximal exercise test provides an accurate measurement of VO_2_ max, there are several limitations. The maximal exercise requires a high level of motivation from the subject and should be performed under medical supervision for older or high-risk subjects who need this test the most [6]. Furthermore, the gas analysis requires expensive equipment and a trained technician to operate the process [7]. In addition, because of the high cost and inconvenience, it is impractical to repeat the maximal exercise test to regularly monitor VO_2_ max.

Several estimation models have been developed to estimate VO_2_ max. Some of these models have developed a submaximal exercise protocol in an attempt to overcome the limitation of the maximal test [8-10]. Submaximal models obtain exercise-related data through a specified exercise protocol, such as shuttle run, and build estimation models along with other anthropometric features. Although submaximal models have overcome some of the limitations of the GXT, they still require trained personnel to conduct the submaximal test, and familiarity with the exercise protocol could affect the results of the test [11], making it unsuitable for regular VO_2_ max monitoring. There are other estimation models that do not involve an exercise protocol [12,13]. These models estimate VO_2_ max by collecting data from physical activity and heart rate (HR) from daily life and using the relationship between the collected data and VO_2_ max. Although these methods are more suitable for regular VO_2_ max measurement, they are time-consuming (requiring a week of data collection) and use multiple devices, making it uncomfortable for application in daily life.

In our previous study [14], we developed a nonexercise VO_2_ max estimation model using a new feature, maximum activity energy expenditure (aEEmax), which was calculated using activity energy expenditure and HR. Using aEEmax, we were able to build an accurate estimation model. However, aEEmax and our previous model were validated only in homogenous subjects, young Asian males. Furthermore, the device was worn on the chest, which might cause discomfort when used in daily life.

The aim of this study was to overcome the limitations of our previous study by using a wristwatch-type fitness tracker with various groups in terms of age and sex. We also sought to develop a new VO_2_ max estimation model using aEEmax and the slope between physical activity and HR as new features, which could be applied to daily life data collected from a single convenient device worn on a wrist with a relatively short estimation time.

## Methods

### Participants

A total of 240 participants were recruited for this study. All participants completed the Physical Activity Readiness Questionnaire and health evaluation, including medical history related to cardiovascular disease, hypertension, and/or diabetes. Only participants without such medical history were included for this study. There were a total of 6 groups, divided according to age (20 to 35, 36 to 50, and 51 to 65 years) and sex, and there were 40 subjects for each group. Subjects who failed to achieve VO_2_ max were excluded from the study. The achievement of VO_2_ max was defined by accomplishing at least 2 of the following 3 criteria: a respiratory exchange ratio reaching >1.2, plateau of VO_2_ despite increasing work load, or self-reported volitional fatigue [15]. Participants who did not wear the device for 3 days or participants with data loss were also excluded. A total of 49 subjects were excluded because of failure to achieve VO_2_ max or unappropriated data collection. The characteristics of the participants are shown in Table 1.

**Table 1:** Subject characteristics.

Characteristics	Male, mean (SD)			Female, mean (SD)		
	20-35 years (n=34)	36-50 years (n=26)	51-65 years (n=30)	20-35 years (n=36)	36-50 years (n=35)	51-65 years (n=30)
Height (cm)	174.3 (5.6)	172.6 (6.1)	167.1 (4.9)	161.9 (5.4)	160.0 (5.2)	155.3 (4.8)
Weight (kg)	73.9 (8.0)	74.7 (9.7)	67.0 (6.1)	55.8 (7.3)	60.6 (6.1)	56.2 (6.1)
Percent body fat	20.4 (5.2)	24.8 (4.9)	24.2 (5.1)	29.4 (6.7)	33.9 (4.9)	33.8 (5.4)
aEEmax^a^ (kcal/kg/h)	141.0 (14.6)	123.7 (15.1)	111.2 (11.0)	112.5 (15.8)	107.7 (12.1)	102.0 (10.3)
Slope (kcal/kg/h/bpm)	1.10 (0.14)	1.04 (0.13)	1.04 (0.12)	0.92 (0.17)	0.98 (0.14)	0.96 (0.13)
VO_2_ max^b^ (mL/kg/min)	42.3 (3.6)	39.9 (3.5)	38.1 (4.6)	35.3 (3.5)	31.4 (4.1)	30.5 (3.9)

^a^aEEmax: maximal activity energy expenditure.

^b^VO_2_ max: maximal oxygen uptake.

### Anthropometrics

Body mass and height were measured using a medical scale with a stadiometer (BSM330; InBody). Body mass was measured to the nearest 0.1 kg, and height was measured to the nearest 0.1 cm. Percent body fat was measured using a bioimpedance analysis (InBody720; InBody) to the nearest 0.1%.

### Measurement of Maximal Oxygen Uptake

The reference VO_2_ max value was measured using the modified Bruce protocol. The equipment used in the modified Bruce protocol includes a respiration gas analyzer (Vmax Encore System; CareFusion) and an aerobic exercise test system (CASE v6.61; GE Healthcare). Standard 12-lead electrocardiogram (ECG), blood oxygen saturation, and blood pressure were measured throughout the procedure.

Before performing the modified Bruce protocol, the baseline physiologic measures for all devices used were measured in a resting state for 5 min and subsequently in a standing position. The modified Bruce protocol was performed immediately after the baseline measurement. The treadmill’s velocity and slope increased at 3-min intervals until the subject reached VO_2_ max.

### Experimental Methods

Participants wore a Fitbit (Fitbit Charge; Fitbit) on the left wrist for 3 consecutive days. From our previous study, we have shown that a minimum of 15 hours of physical activity data are required to acquire aEEmax [14]. In total, 3 consecutive days, regardless of weekday or weekend days, were shown to be enough to obtain the data needed based on the study. The Fitbit simultaneously measured HR and daily physical activity in terms of metabolic equivalent (kcal/kg/h), which was an expression of energy expenditure of activities.

The participants removed the sensor during sleep or showering. Data obtained from the sensor were retrieved after the experiment via the internet. The Fitbit returned HR and metabolic equivalent data for every 1 min.

### Signal Processing

Moving average filter was applied to both HR and physical activity data. After filtering the data, only data points at which both HR and physical activity data increased were selected as a period of physical activity for further processing. This was done by differentiating the data and selecting where both differentiated data were positive.

**Figure 1:**
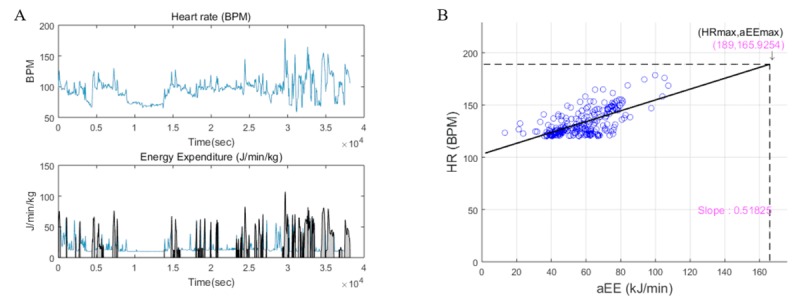
(A) HR and aEE data from Fitbit. The shaded area indicates the period of data where both HR and aEE are increasing. (B) Scatter plot between selected periods of aEE versus HR of a representative subject. Data where HR was less than 120 bpm were removed to select the data where HR and aEE had a linear relationship. aEEmax is defined as the intersection between the interoperation line and HRmax, and the slope is the slope of the interpolation line. aEE: activity energy expenditure; aEEmax: maximal activity energy expenditure; BPM: beat per minute; HR: heart rate; HRmax: maximum heart rate.

Figure 1 shows the HR (upper graph) and physical activity (lower graph) for a representative participant’s filtered data over time. The shaded area under the physical activity curve represents the periods of increasing HR and physical activity. The scatter plot for HR versus physical activity is shown in Figure 1. A simple linear regression was performed between HR and physical activity to estimate aEEmax. The part of data where HR was greater than 120 beats/min was selected to remove the data in which the relationship between HR and physical activity was nonlinear [16]. The maximal HR was calculated as 200–age×0.67 for women and 216–age×0.93 for men [17]. The physical activity value of the point of intersection between the maximum HR and the regression line was defined as the aEEmax. The slope of the regression line was also used as a feature to estimate VO_2_ max; hereafter, it will be referred as the *slope*. After the calculation of aEEmax and the slope, a multiple linear regression model was developed with aEEmax and anthropometric values to estimate VO_2_ max.

### Statistical Analysis

Pearson correlation coefficient was calculated between the independent variables (age, percent body fat, height, gender, aEEmax, and slope) and the measured VO_2_ max. The regression model for estimating VO_2_ max was evaluated with the coefficients of determination (adjusted *R*^2^) and absolute SE of the estimate (SEE). The predicted residual error sum of squares (PRESS) statistic method was selected for cross-validation of the model [18]. The PRESS statistic is a cross-validation method calculating the error for each case by excluding a case each time from generating the estimation model and applying the model to the excluded case. The PRESS adjusted *R*^2^ (R^2^p) and the PRESS SEE (SEEp) were calculated as 1 – (PRESS/SS) and . The model was further validated by dividing it into different subgroups and calculating the constant error (CE) for each group. The standard for recruiting participants was to retain diversity. However, we wanted to observe CE based on age, sex, and VO2max level. The median value for age and VO2max was chosen to divide the groups. All signal processing, cross-validation, and statistical analyses were performed using MATLAB (MATLAB2017a; MathWorks).

### Ethics Statement

This study protocol was reviewed and approved by the Institutional Review Board of the Seoul National University Hospital (IRB No. 1505-022-669). Written informed consent was submitted by all subjects when they were enrolled. This study followed the Helsinki Declaration.

## Results

The general characteristics of all subjects are summarized in Table 1. The average value for age, weight, height, and percent body fat for excluded male subjects were 42.0, 72.8 kg, 171.0 cm, and 25.0% respectively. For excluded female subjects, the average values were 41.7, 56.5 kg, 158.4 cm, and 31.6%, respectively. A student *t* test was performed to compare the *P* value for age, weight, height, and percent body fat between included and excluded subjects. For male subjects, the *P* values were .646, .937, .883, and .026, respectively. For female subjects, the *P* values were .936, .415, .663, and .497 respectively.

The Pearson correlations between VO_2_ max and selected features are shown in Table 2. The Pearson correlations between selected features are also summarized. The correlations between VO_2_ max and independent variables were all statistically significant (*P*<.001 for all). The independent variable that showed the highest correlation was sex, with a correlation of .675. The lowest correlation for the model was the *slope*, with a correlation of .237.

**Figure 2:**
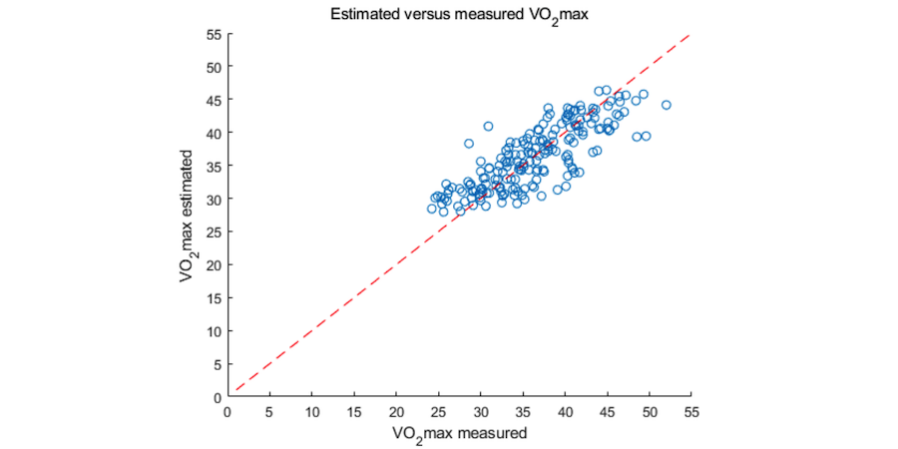
The correlation between estimated and measured VO2max value for all subjects (N=191). The red solid line is the identity line of the measured and estimated VO2max. VO2max: maximal oxygen uptake.

The multiple linear regression analysis for the model is shown in Table 3. The scatter plot for measured VO_2_ max versus predicted VO_2_ max is shown in Figure 1. The *R*^2^ for the model was 0.651, and the SEE was 3.518 mL/kg/min. As shown in Table 3, the decrease in *R*^2^ and the increase in SEE were small for the cross-validation result of the PRESS method. *R*^2^ decreased by 0.032 and SEE increased by 0.148 mL/kg/min. The scatter plot of the multiple linear regression for the model is shown in Figure 2.

The model was further validated by dividing the groups into various subgroups and calculating the CE and SD. Each subgroup was divided into 2 groups according to age, sex, and measured VO_2_ max. The results are shown in Table 4. The CEs were positive for the younger group and negative for the older groups. The CE for the younger group was 0.024 mL/kg/min. The CE for the older group was −0.021 mL/kg/min. The CEs were all positive for the subgroups divided according to sex. The CE was positive for individuals with high VO_2_ max and negative for individuals with low VO_2_ max. However, as shown in Table 4, CE values were low for all subgroups, indicating that our model does not overestimate or underestimate VO_2_ max.

**Table 2:** Correlation matrix between VO_2_ max and independent variables.

Independent variables	VO_2_ max^a^	Age	Height	Sex	Percent fat	aEEmax^b^
Age (years)	−0.372^c^	—	—	—	—	—
Height	0.372^c^	−0.329	—	—	—	—
Sex	0.675^c^	−0.015	0.517	—	—	—
Percent fat	−0.652^c^	0.202	−0.423	−0.620	—	—
aEEmax	0.503^c^	−0.473	0.397	0.496	−0.340	—
Slope	0.237^c^	−0.016	0.193	0.369	−0.130	0.830

^a^VO_2_ max: maximal oxygen uptake.

^b^aEEmax: maximal activity energy expenditure.

^c^*P*<.001.

**Table 3:** A multiple regression nonexercise model for estimating VO_2_ max (maximal oxygen uptake; mL/kg/min).

Independent variables	Fitbit model	
	Coefficient	Beta^a^
Constant	63.262	—^b^
aEEmax^c^ (kcal/kg/h)	0.027	.082
Slope (kcal/kg/h/bpm)	−1.776	−.045
Percent body fat	−0.242	−.296
Age (years)	−0.150	−.321
Sex	3.264	.548
Height (cm)	−0.09	−.166
*R* ^d^	0.807	—
SEE^e^ (mL/kg/min)	3.518	—
*R*_P_^f^	0.787	—
SEE_P_^f^ (mL/kg/min)	3.667	—

^a^Beta is the normalized coefficient of the model.

^b^—: not applicable.

^c^aEEmax: maximal activity energy expenditure.

^d^*R* is the Pearson correlation.

^e^SEE: SE of estimate.

^f^*R*_P_ and SEE_P_ are the cross-validated results of the model.

**Table 4:** Constant error and SD for each subgroup. Subjects aged older than 40 years are considered the old group, and subjects with a VO_2_ max higher than 36 mL/kg/min are considered as the high VO_2_ max group.

Group	n (%)	CE^a^	SD
Female	101 (52.9)	−4.397e-15	4.341
Male	90 (47.1)	3.000e-15	4.484
Old	101 (52.9)	−0.021	5.587
Young	90 (47.1)	0.024	5.180
High VO_2_ max^b^	77 (40.3)	1.535	3.565
Low VO_2_ max	114 (59.7)	−1.488	3.232

^a^CE: constant error.

^b^VO_2_ max: maximal oxygen uptake.

## Discussion

In this study, we developed VO_2_ max estimation models with data collected from the commercially available device, Fitbit, which could estimate VO_2_ max conveniently from daily life. The commercial device used in this study provided data on physical activity, along with its physiological response (HR). Even though some previous studies have reported that Fitbit does not always generate accurate data [19,20], our method for estimating VO_2_ max does not depend on the absolute value of each data point. It rather depends on the trend of a large set of data points and would balance the inaccuracy of a single data point. Even though Fitbit data were biased under certain conditions, a large number of data collected from daily life would minimize this bias. This characteristic of our 2 novel features would only need calibration to be applied with other hardware and processing methods. Previous studies reported that lower HR was not linearly correlated with physical activity [16]. It is also known that heterogeneous recovery of HR after physical exercise does not have a clear correlation with VO_2_ max [21]. Therefore, in an attempt to select the data during physical activity, we have selected periods of data where HR is greater than 120 beats/min and where both HR and physical activity increased at the same time to calculate aEEmax and the slope. By calculating 2 features from the linear relationship between HR and energy expenditure, our model would estimate VO2max accurately without any protocol or training.

In our previous study [14], aEEmax and our estimation model were validated with a homogenous group of young healthy Asian males using aEEmax and BMI (VO2max=0.192 x aEEmax – 0.708 x BMI). In this study, we have validated aEEmax and the model with a large pool of subjects, including both men and women aged from 20 to 65 years. The validation of the model was performed by cross-validation with the PRESS method and by calculating the CE of the subgroups. The cross-validated result shows that the validation sample fitted well with the model with little error. Furthermore, the CE for the model shows that our methodology did not significantly overestimate nor underestimate VO_2_ max for all subgroups, whereas other studies [12,22] have reported significant overestimation and underestimation with both highly and poorly fitted individuals.

To the best of our knowledge, this study is the first to provide a VO_2_ max estimation model with a commercially available device on a wrist without any specific protocol. Tönis et al used a submaximal exercise protocol to estimate VO_2_ max [23]. Polar Electro Oy Inc developed a nonexercise protocol, Polar Fitness Test, and devices to estimate VO_2_ max. However, Esco et al [24] reported that the Polar Fitness Test had low accuracy for estimating VO_2_ max when it was tested with one of its own products. Altini et al [25] developed a nonexercise estimation model for VO_2_ max with data collected from daily life; however, they used an ECG necklace with a wet electrode attached to the chest and stomach, which could cause an inconvenience when used in daily life. Our method allows individuals to measure their VO_2_ max on a wrist without the requirement for any electrode attachment. In addition, although other smartwatches, including Fitbit, require a specific protocol, such as running for at least 10 min on flat terrain, our protocol does not require any protocol and allows for easy monitoring of physical fitness.

Instead of using the absolute value of HR or physical activity data, we developed 2 new features to portray physical fitness. The change in HR for a given physical activity differs depending on the physical fitness of a subject [8]. Thus, the slope between HR and physical activity would be smaller and aEEmax would be larger for a subject with higher VO_2_ max. As shown in Table 2, aEEmax has a moderate correlation with VO_2_ max, supporting our hypothesis. Features based on physical activity can easily fluctuate depending on change in the short-term lifestyle of the subject during the period of data collection. Those features would be vulnerable to a sudden increase or decrease in the amount of physical activity. On the contrary, aEEmax and the slope represent the relationship between physical activity and HR and thus would be less affected by a sudden short-term change in physical activity. In addition, aEEmax and slope have been shown to be applicable to a wide range of subject ages and different genders. Other studies [26-28] have been validated with a relatively homogenous group compared with this study. The percent body fat used in this study was obtained using a professional bioimpedance analyzer. However, we have used general percent body fat which did not necessarily require a professional analyzer. There are products available, such as an AURA device, and ongoing studies about measuring body fat percentage from the wrist [29]. These efforts will make the measurement of percent body fat more accessible to the public.

There are limitations to this study. First, our methodology needs to be validated with more devices worn on a wrist. There are many commercially available devices that provide physical activity and HR data. To provide a more generalized VO_2_ max estimation method, it is important to prove device independency of our method. In addition, this study was conducted with healthy subjects who were not taking any medication that might affect the HR. Another limitation of this study was error with maximal HR calculated from a basic population-derived formula. A more accurate method for calculating maximal HR could increase the accuracy of our model. A future study could include subjects who are on cardiac-related medication. Additionally, in our previous study, we have shown that 900 min of data were enough to calculate aEEmax; however, it would be worthwhile to observe change in the correlation coefficient of aEEmax for a longer period of time.

In summary, we have developed a new estimation model for VO_2_ max using novel features, aEEmax and the slope between physical activity and HR, along with other anthropometric variables. The new features represent the relationship between physical activity and its physiological response. The high correlation between VO_2_ max and aEEmax is in agreement with our previous study and supports our hypothesis. Our model requires data from only 3 days of daily life, without any specific exercise protocol. This hypothesis was validated with a diverse and large number of participants based on age and sex. Furthermore, all material required for our study is available in the conventional market as fully built products. The result of this study allows individuals to measure their VO_2_ max conveniently in their daily life without any burden of an exercise protocol and allows them to easily monitor physical fitness.

## Abbreviations

aEEmax: maximal activity energy expenditure
CE: constant error
CRF: cardiorespiratory fitness
ECG: electrocardiogram
GXT: graded exercise test
HR: heart rate
PRESS: predicted residual error sum of square
SEE: SE of estimate
VO2max: maximal oxygen uptake
